# Evaluation and Dissemination of a Checklist to Improve Implementation of Work Environment Initiatives in the Eldercare Sector: Protocol for a Prospective Observational Study

**DOI:** 10.2196/16039

**Published:** 2020-05-13

**Authors:** Charlotte Diana Nørregaard Rasmussen, Helene Højberg, Anne Konring Larsen, Pernille Kold Munch, Richard Osborne, Lydia Kwak, Irene Jensen, Laura Linnan, Marie Birk Jørgensen

**Affiliations:** 1 The National Research Centre for the Working Environment København Ø Denmark; 2 Faculty of Health, Arts and Design Swinburne University of Technology Hawthorn Australia; 3 Unit of Intervention and Implementation Research for Worker Health Institute of Environmental Medicine Karolinska Institute Stockholm Sweden; 4 Carolina Collaborative for Research on Work & Health Gillings School of Global Public Health University of North Carolina Chapel Hill, NC United States; 5 Health and Safety Municipality of Copenhagen Copenhagen Denmark

**Keywords:** RE-AIM, implementation, workplace, digital

## Abstract

**Background:**

To measure sustainable improvements in the work environment, a flexible and highly responsive tool is needed that will give important focus to the implementation process. A digital checklist was developed in collaboration with key stakeholders to document the implementation of changes in eldercare sector workplaces.

**Objective:**

This paper describes the study protocol of a dissemination study that aims to examine when, why, and how the digital checklist is spread to the Danish eldercare sector following a national campaign particularly targeting nursing homes and home care.

**Methods:**

This prospective observational study will use quantitative data from Google Analytics describing use of the checklist as documented website engagement, a survey among members in the largest union in the sector, information from a central business register, and monitoring of campaign activities. The evaluation will be guided by the five elements of the RE-AIM framework: reach, effectiveness, adoption, implementation, and maintenance.

**Results:**

The study was approved in June 2016 and began in October 2018. The campaign that is the foundation for the evaluation began in 2017 and ended in 2018. However, the webpage where we collect data is still running. Results are expected in 2020.

**Conclusions:**

This protocol provides a working example of how to evaluate dissemination of a checklist to improve implementation of work environment initiatives in the eldercare sector in Denmark. To our knowledge, implementation in a nationwide Danish work environment has not been previously undertaken. Given that the checklist is sector-specific for work environment initiatives and developed through systematic collaboration between research and practice, it is likely to have high utility and impact; however, the proposed evaluation will determine this. This study will advance dissemination research and, in particular, the evaluation of the impact of these types of studies. Finally, this study advances the field through digital tools that can be used for evaluation of dissemination efforts (eg, Google Analytics associated with website) in the context of a rigorous research design activity.

**International Registered Report Identifier (IRRID):**

DERR1-10.2196/16039

## Introduction

Currently many countries are facing shortages of health care workers, and this is jeopardizing capacity to deliver residential care services [1]. The shortage of health care workers is associated with high rates of sickness absence, turnover, and early retirement among this professional group [2]. It is well known that high physical and psychosocial work demands are important risk factors for long-term sickness absence, turnover, and early retirement from the eldercare sector [3,4]. Thus, increasing and sustaining the eldercare workforce demands urgent attention. Several initiatives, including complex multilevel interventions, have been introduced to improve the work environment of eldercare workers during the past decades in Denmark and other countries [5-8]. However, despite availability of extensive research and policy efforts, employees in the eldercare sector have only experienced limited improvements in the work environment.

A reason for the lack of improvement in the work environment in this setting may be that the eldercare sector is continuously changing through care regimes, political reforms, and high turnover rates [9]. Such changes affect the stability of the organizations and may also challenge implementation of new knowledge and work environment policies or initiatives [9]. The work environment can be considered a moving target with continuously changing terms and starting points [10]. Therefore, interventions for such a moving target must be flexible and highly responsive to the changing needs in the work environment and facilitate the implementation process [10]. Therefore, we decided to collaborate with stakeholders from the target group to collect information about how to efficiently implement changes in the work environment in the eldercare sector [10]. The knowledge base was condensed to the Hitting the Moving Target framework, which summarized 11 components to consider in order to succeed with implementation [10]. The framework targets both managers and employees in the eldercare sector. From recommendations from the target group, the 11 components from the Hitting the Moving Target framework were used to develop an implementation tool in the form of a digital checklist containing the 11 concepts.

Another reason for the lack of improvement in the work environment in this sector may be the challenge of translation of policies and research knowledge into practice [11]. Many factors can influence whether the translation of research knowledge into practice is successful and whether policies or evidence-based practices are accepted and used by the target users [12]. Dissemination of research findings is an important step to bridge the gap between research and practice. Effective dissemination strategies include formative research to customize dissemination strategies to fit audience needs and preferences [13]. Distribution strategies should focus on ensuring that messages and materials from research reach intended audiences by use of multicomponent dissemination strategies (eg, mailings, websites, publications, webinar or in-person presentations, interpersonal connections, and mass media, among others) [13,14]. To be most effective, distribution should engage the channels that intended audiences already trust and access for information [13]. Previously, national campaigns have been used to reach a large proportion of the population for reducing musculoskeletal disorders [15,16]. But such campaigns are expensive and need to be well planned. In addition, the packaging and communication used to disseminate evidence-based knowledge determines the dissemination success [17]. Different approaches have been attempted to overcome this challenge. For example, in Sweden, guidelines aimed at improving the psychosocial work environment have been coproduced with practitioners [18]. Still, there is a shortage of knowledge on how to optimize dissemination, and the reach and effect of such initiatives is difficult to evaluate and rarely investigated.

This paper is the dissemination protocol that examines when, why, and how the checklist is spread to the Danish eldercare sector, in particular nursing homes and homecare. The protocol presents both the process of developing the digital checklist, planning the dissemination strategy (ie, the communication of the checklist through a national campaign), and the evaluation plan. Specifically, the protocol aims to investigate the adoption, reach, implementation, maintenance, and effectiveness of the checklist after a national campaign to improve implementation of work environment initiatives among eldercare workers in Denmark. The following four research questions will be investigated:

1. How many Danish eldercare workplaces use the checklist and what characterizes those who do from those who don’t? (adoption)
2. Across Danish eldercare workplaces, what proportion of eldercare workers know about the campaign and what characterizes those who do from those who don’t? (reach and representativeness)
3. Among the users, how is the checklist used and for what purposes? (implementation and maintenance)
4. Is the work environment practice improved among users of the checklist compared with nonusers? (effectiveness)

## Methods

### Study Design

This is a prospective observational study using a range of quantitative data collection approaches to accomplish the study aims. The study is described according to the Standards for Reporting Implementation Studies (StaRI) statement.

### Study Setting and Population

The study setting is the eldercare sector in Denmark, and more specifically, selected nursing homes and homecare settings. In Denmark, there are approximately 5000 workplaces within the eldercare sector that employ about 100,000 eldercare workers.

### Ethics Approval and Consent to Participate

According to Danish law as defined in Committee Act §2 and §1, the study described should not be further reported to the local ethics committee. The data use is approved by the Danish Data Protection Agency. According to Danish law, questionnaire- and register-based studies do not need approval by ethical and scientific committees or informed consent by participants.

### Development of the Digital Checklist (Dissemination Object)

The translation from Hitting the Moving Target framework to a digital checklist was conducted in close collaboration with relevant partners. We involved two groups of stakeholders: (1) a practice-based research team (PBR) team and (2) a municipality team. They functioned as codevelopers with a central role in operationalizing the 11 implementation components from the Hitting the Moving Target framework into the checklist. The PBR team group consisted of 10 stakeholders with representatives from the largest union in the nursing home sector, Local Government Denmark, which is the central organization of all Danish municipalities, the Danish Work Environment Authority, the Knowledge Center for Work Environment, and the Sector-Specific Work Environment Community Organization for Public and Welfare workplaces. The municipality-based stakeholder group consisted of 5 work environment consultants employed in 4 different Danish municipalities, and this group helped us in the development, especially in making the checklist adaptable to existing practices and structures. As an example, the municipality-based stakeholder group helped us decide that the checklist should be directed at the Occupational Health and Safety (OHS) groups at the workplaces, which are groups consisting of both managers and employees with specific tasks related to OHS.

**Figure 1:**
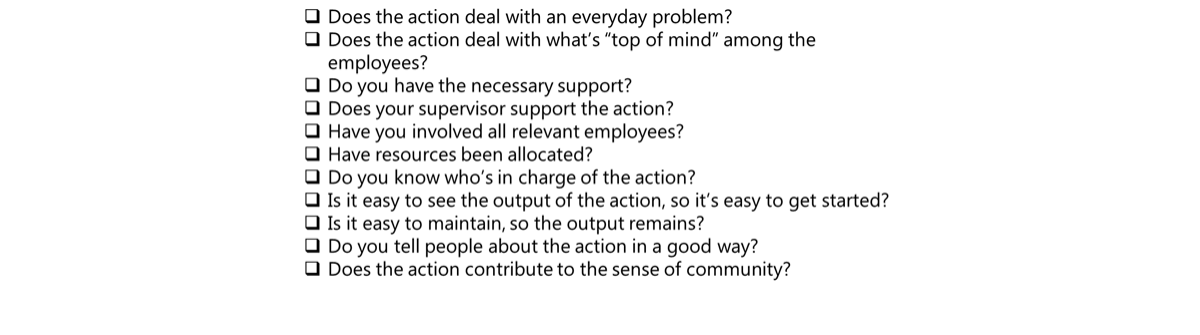
The wording of the checklist (translated from Danish). Before going through the checklist, the respondents had defined an action (free of choice, eg, “we wish to implement a new assistive device”) they wanted to focus on to improve the work environment.

The feasibility of the checklist was assessed through cognitive interviews with a range of intended beneficiaries, both employees and managers in the eldercare sector (primarily those involved in OHS groups). The prototype of the Web-based checklist was then included in 40 tests conducted with employees, mainly OHS representatives at different nursing homes, to fit the digital solution to the user needs and make sure they could use the checklist as intended. The final 11 checkpoints can be seen in Figure 1.

### Content and Use of the Web-Based Checklist

**Figure 2:**
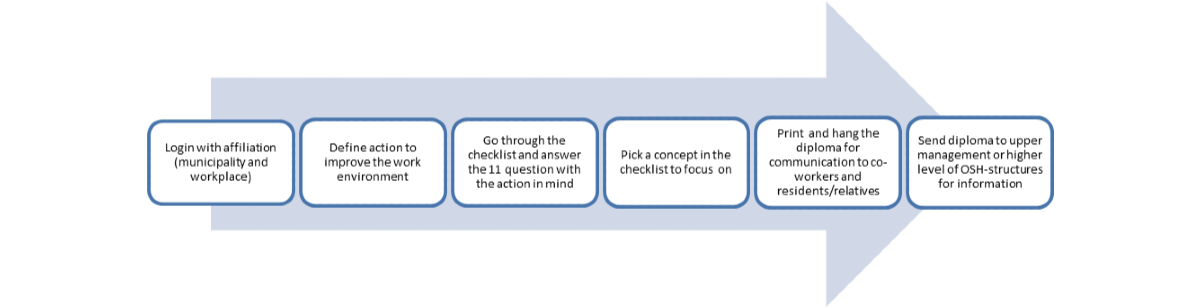
Overview of the steps on the Web-based checklist.

The checklist is an interactive digital platform that can be assessed through a website. Users (primarily the OHS groups) can use it in their work environment practice when implementing new routines, projects, or initiatives (termed *actions* in the checklist) to improve the work environment. The steps in the process of using the checklist are described in Figure 2. The user has to log in with their affiliation and then define the action (free of choice) they want to implement. The next step is to go through the checklist and pick one concept in the checklist that they want to focus on. Working with defined actions to support implementation is a task that the OHS groups already have in their portfolio to maintain a good work environment. After having gone through all the points of the checklist they get a result that indicates how many facilitators for implementation are in place, and they get information about the concept to focus on. It is possible to print a diploma that shows this and place it visibly at the workplace and automatically send an email to communicate the actions to others (ie, coworkers and upper management). In addition, it is possible to get tips for implementation on the website [19].

### Dissemination Strategy

#### National Campaign

The aim with the campaign was to increase awareness of the existence of the checklist. The campaign was developed with our PBR and municipality teams. An initial workshop was held to identify central target users when disseminating the checklist to the eldercare sector. Through persona analysis, we characterized all potential users working with or within the eldercare sector and made a description of their role in the work environment, and thereby we were able to describe who would be the most relevant target group for the checklist. This workshop highlighted the importance of targeting the checklist directly to those who work within the work environment (eg, the OHS groups) at the workplaces. We planned the campaign to be nationwide and primarily driven through network on social media, websites, through newsletters, magazines, conferences, letters, and a campaign film by the researchers and stakeholders (especially the PBR team). The campaign was planned to run for 1 year (from September 2017 to September 2018).

#### Campaign Materials and Methods

The campaign materials included paper and digital elements. Paper elements included printed versions of the checklist such as postcards and letters, which were sent to the administrative departments of all municipalities and all identified home care units and nursing homes in Denmark informing about the new checklist. The digital elements included a short campaign movie, small instruction movies, and newsletters. We produced a campaign movie aimed at increasing awareness about the new checklist. The movie was distributed via social media (eg, Facebook, LinkedIn, and Twitter), and we aimed to create awareness of the newly developed checklist among the entire target population in the eldercare sector. Additionally, we produced small instruction movies showing how to use the checklist. Both were uploaded on the same webpage [20].

#### Presentations and Dissemination Partners

The researchers presented the checklist whenever possible at conferences and workshops where the target group was present. Furthermore, researchers presented the checklist for consultants working in this industry—for example, to consultants employed at a central position in the municipality or physiotherapists or occupational therapists working at one or more nursing homes. We focused on building a network with central work environment representatives in the municipalities of Denmark, emphasizing the close collaboration between researchers and practitioners. This enabled exchange of information from the municipalities about the work environment and for us to inform about the checklist and how to use it.

The stakeholders (especially our PBR team) functioned as dissemination partners. They referred to the checklist on their respective websites, Facebook pages, in magazines, in newsletters, etc. The PBR team also functioned as ambassadors for disseminating the checklist (eg, when visiting the nursing homes). Furthermore, the entire corps of inspectors from the Danish Work Environment Authority (N-90), who visit and inspect workplaces in Denmark regarding the work environment, were trained in the checklist and informed about the checklist before going out to the workplaces. Additionally, we established a partnership with work environment consultants connected to the eldercare sector. They were informed about the checklist when relevant—for example, when talking with the OHS groups about potential initiatives for improving the work environment and how to succeed with their initiatives.

### Evaluation

This study aims to investigate the dissemination and effectiveness on work environment improvements of a checklist to improve implementation of work environment initiatives among eldercare workers in Denmark. A commonly used framework in the evaluation of implementation research is the RE-AIM (reach, efficacy/effectiveness, adoption, implementation, and maintenance) framework [21-24]. RE-AIM guides areas to consider when seeking to evaluate the potential public health impact of a program and consists of the 5 dimensions, reach, efficacy/effectiveness, adoption, implementation, and maintenance.

**Figure 3:**
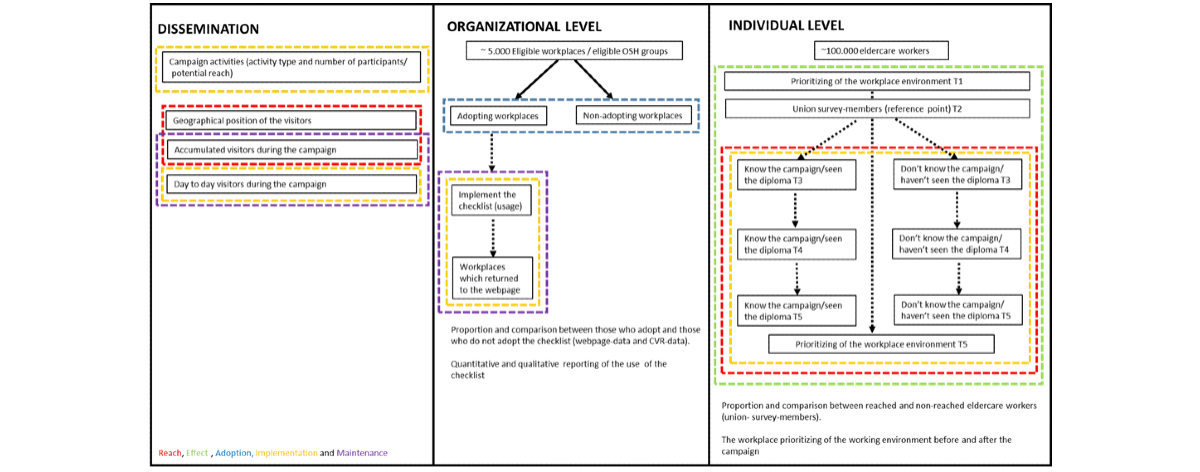
Overview of the evaluation. The different evaluation components are color-coded according to the RE-AIM framework.

In this dissemination study, the indicators of reach, implementation, and maintenance are somewhat overlapping and may be used as indicators of more than one evaluation component. This is because the intervention can be considered as both the campaign, the checklist, and the action plan at the workplace and thus reach and implementation can occur at several levels (ie, societal, organizational, and individual level), and we aim to describe all levels in this study (see Figure 3).

### Data Collection

**Figure 4:**
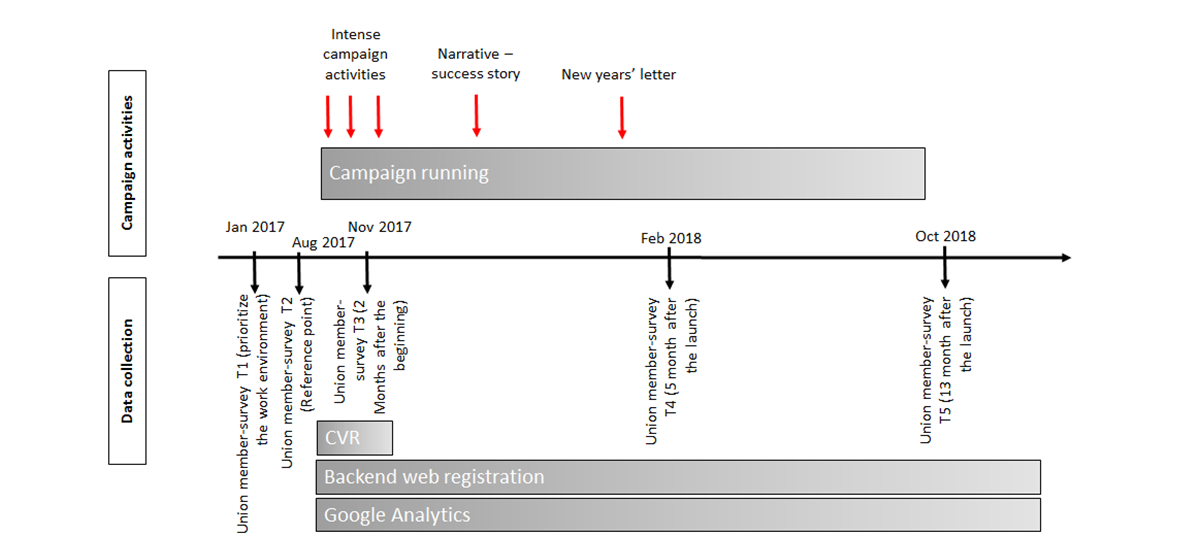
Overview of campaign activities and data collection.

We will use multiple data sources including data on campaign activities, data from the Central Business Register (CVR), data from Google Analytics, data regarding activity on the project website (use of the checklist), and data from a survey among members in the largest union in the industry. In Figure 4, the campaign (activities) and the data collection are illustrated.

#### Central Business Register

The CVR contains information about all registered workplaces in Denmark. Factors available in the CVR are size (number of employees in intervals), type of workplace (nursing home, home care, hospital, etc), age (start-up date of company/workplace), and geographical position of the workplace (ie, municipality).

#### Google Analytics

The website [19] was associated with a Google Analytics account. Information on day-to-day visits and month-by-month geographical data on city level will be downloaded.

#### Online Checklist Website

From the backend of the website, we will collect user-specific information from visitors, defined actions to improve the work environment, and answers to the checklist.

#### Union Survey

The largest trade union for eldercare workers in Denmark organizes approximately 180,000 members primarily in the public sector. The union has more than 90,000 employed members engaged in social and health sector. Members belonging to the social and health sector can voluntarily sign up as a survey member to receive a questionnaire 4 to 6 times a year about work environments and other work-related topics. Union members can register and drop out as they want. The union survey is sent to approximately 7500 survey members each time.

The data sources above will be used to answer the research questions according to the RE-AIM framework. Multimedia Appendix 1 shows each of the components of the RE-AIM framework and their definition. In addition, the appendix shows the specific research question(s) related to each component and their respective data source and operationalization.

### Statistical Analysis

Using descriptive statistics, we will compare adopting (workplace that use the checklist) and nonadopting workplaces in terms of the number of employees, type of workplaces, and age and geographical position of the workplaces. To report reach, we will use descriptive statistics to compare gender, age, manager, position of trust, employer, and workplace of respondents with knowledge of the campaign and those not reached by the campaign. In addition, we will test for differences in adopters and nonadopters and in reached and nonreached, respectively, by *t* test or analysis of variance.

Effectiveness will be evaluated by comparing the change in the prioritization of the work environment efforts experienced by respondents from before the campaign until after the campaign. Analyses regarding the effectiveness will be performed after 12 months of campaign by means of analysis of covariance comparing employees with knowledge of the campaign and those without knowledge of the campaign. We will test whether it is relevant to control for confounders such as age and gender.

## Results

The study started in September 2017 with the 1-year campaign. The main data collection was completed by September 2018, but data collection through the website is ongoing. Dissemination of results is expected in early 2020.

## Discussion

### Summary

This paper presents the protocol for the evaluation and dissemination of a checklist to improve implementation of work environment initiatives in the eldercare sector in Denmark. To our knowledge, this has not previously been undertaken in a Danish work environment context, and it is the first nationwide sector-specific checklist for implementing work environment initiatives. Also, evaluation of which checklist points have been most frequently (or rarely) ticked may give valuable information regarding which implementation challenges workplaces easily handle and which challenges they postpone.

The project will expand the understanding of the determinants of implementation and dissemination success and failure. New knowledge will be generated on industry-specific dissemination, which potentially can be used in industries other than nursing homes. It is hoped that the planned fine-grained evaluation outcomes will provide practical information that will give managers and government agencies well-tested tools and comprehensive process insights that with enable interventions to be more quickly and more effectively implemented to generate improved work environments and alleviate some of the shortages in the eldercare workforce.

### Strengths and Limitations

A limitation in the evaluation of the impact of the checklist is that use is measured only through the website and not the potential use outside this setting. For example, the postcards with a print of the checklist given to workplaces can be used instead of logging in to the website checklist. Therefore, the evaluation will likely not cover the full dissemination and may actually underrepresent uptake of the checklist. Further, we have observed that some employees in eldercare may have limited access to computers during work hours. On the other hand, tablets and laptops are often present at the workplaces and Web-based work environment and safety systems are becoming a more regular practice. A limitation to the study is that we cannot generalize beyond eldercare. It is also a limitation that the data are self-reported and contained nonvalidated information. However, a strength is the use of multiple data sources and new digital data sources.

### Conclusion

To have a wide and lasting impact, tools often need to be adapted to or reconstructed within practice settings. Consequently, to maximize the utility of our model and the resulting implementation checklist, extensive and systematic involvement of stakeholders was undertaken. Importantly, we also used this codesign process to develop the dissemination strategy. We intended that the ownership generated in key stakeholders would assist to ensure the development of a fit-for-purpose checklist and dissemination strategy and therefore generate strong positive outcomes. There is an ever-increasing demand for research to make a practical difference in policy and in society in general, but many products and programs fail deliver such outcomes. Given the methods used to develop our interventions and the approach to evaluation, we expect that the data generated from this study will generate novel insights into how to generate real-world impacts at scale. In conclusion, this study will advance dissemination research and, in particular, the evaluation of the impact of these types of studies. Finally, this study advances the field through digital tools that can be used for evaluation of dissemination efforts (eg, Google Analytics associated with website) in the context of a rigorous research design activity.

## Appendix

Multimedia Appendix 1 Overview of evaluation and operationalization of the components in the RE-AIM framework: adoption, reach, implementation, effectiveness, and maintenance.

Multimedia Appendix 2 Funding documentation. Declaration of translation into English from Danish.

## Abbreviations

CVR: Central Business Register
PBR: practice-based research
OHS: Occupational Health and Safety
RE-AIM: reach, efficacy/effectiveness, adoption, implementation, and maintenance
StaRI: Standards for Reporting Implementation Studies
