# Supplementary material for: Evaluation and Dissemination of a Checklist to Improve Implementation of Work Environment Initiatives in the Eldercare Sector: Protocol for a Prospective Observational Study
Source: JMIR Res Protoc. 2020 May 13;9(5):e16039. doi: 10.2196/16039 (PMC7254284; doi:10.2196/16039)
Supplement: Multimedia Appendix 1 [file resprot_v9i5e16039_app1.docx]

Multimedia Appendix 1. Overview of evaluation and operationalization of the components in the RE-AIM framework; adoption, reach, implementation, effectiveness and maintenance.

| **RE-AIM** | **Definition** | **Research question** | **Data source** | **Operationalization** |
| --- | --- | --- | --- | --- |
| **Adoption** | *“The absolute number, proportion, and representativeness of settings and intervention*   *agents (people who deliver the program) who are willing to initiate a program”** | How many Danish eldercare workplaces use the checklist?   What characterizes those who do from those who do not? | Online checklist website and CVR | How many create an account and log into the online checklist website, where is the person located (municipality), what characterize the adopting workplace and non-adopting workplaces (size (number of employees in intervals), type of workplaces (nursing home, home care, hospital etc.) and starting year and geographical position of the workplaces (i.e. regions)) |
| **Reach** | *“The absolute number, proportion, and representativeness of individuals who are willing to participate in a given initiative, intervention, or program”** | Across Danish eldercare workplaces what proportion of eldercare workers know about the campaign and what characterizes those who do from those who don’t | Online checklist website, Google Analytics and union survey | How many unique visitors to the online checklist website (day to day activity, accumulated activity and geographical position) during the campaign, do you know the campaign (yes, no or don’t know)****, where have you heard of the campaign (network, OHS representative, colleagues, the Danish Working Environment Authority, employer/sector association, the Sector-Specific Work Environment Community Organization for Public and Welfare workplaces, trade union (FOA), website/newsletter, conference or similar, flyer, other or don’t know/don’t remember)****, what characterize the reached from the non-reached (age, gender, manager (yes/no), position of trust (OHS representative, employee representative, OHS representative and employee representative or no position of trust), employer/sector (municipality or an independent institution, private/private resident, region, self-employed, state or other/don’t know), workplace (temporary agency, treatment/district psychiatry, home care, social psychiatry, school, rehabilitation, hospital, nursing home, special area, handicap assistant or other), seniority, working hours per week and shift work (day, evening, night, shifting (with/without night)). |
| **Implementation** | “*How closely staff members follow the program that the developers provide. This includes consistency of delivery as intended and the time and cost of the program*”* |  |  |  |
| ***Dose delivered*** | *“The number or amount of intended units of each intervention or each component delivered or provided. Dose delivered is a function of efforts of the intervention providers”(22)* | Dissemination success | Log-book of all dissemination activities, Google Analytics and Online checklist website | Which dissemination action happened to promote the checklist during the one-year campaign (oral presentations at conferences,  social media activities and newsletters etc.) and how did the dissemination action affect the activity on the online checklist website |
| ***Dose received*** | *“The extent to which participants actively engage with, interact with, are receptive to, and/or use materials or recommended resources. Dose received is a characteristic of the target audience and it assesses the extent of engagement of participants with the intervention”(22)* | Workplace implementation - how the checklist is used and for what purposes | Union survey  (individual level)  Online checklist website (Organizational level) | Individual level: Have you seen this diploma at your workplace (yes, no or don’t know)**   Organisational level: (workplace activity at the online checklist website)   - Unique users   - Number of log-in’s per user  - The action they want to implement  - Number of actions per user  - Number of times working to implement the different actions  - Types of implementation challenges (typically non-ticked “action” points in the checklist)  - Types of implementation successes (typically ticked points in the checklist)  - Types of checklist points most frequently ticked from start.   - Type of usage (printed diploma, letter or tips) |
| **Effectiveness** | “*The impact of an intervention on important outcomes*”* | Whether the prioritizing of the work environment has improved among the users of the checklist compared to non-users during the campaign period | Union survey | Does your workplace in general prioritize the work environment (to a very great extent, to a great extent, to some extent, to a small extent, not at all or don’t know)*** |
| **Maintenance** | “*The extent to which a program becomes a part of the routine organizational practices*”* | The long-term and repeated use of the checklist | Online checklist website | How was the checklist used for workplaces that returned to the online checklist website |

*http://www.re-aim.org/about/frequently-asked-questions/
**The question regarding the diploma will also show a picture of the diploma, which is the end-point of using the checklist. Thus, a “yes” to that question indicates that the workplace has used and gone through the entire checklist.
***The effectiveness will be measured by changes in the union members’ perception that their workplaces prioritize the work environment, comparing those who report to have seen the checklist (users) with those who report to have not seen the checklist (non-users) during the one year of the campaign.
****Union-survey-members will be asked the question just before the campaign starts (to establish a reference point), just after the beginning of the campaign (after two months), after 5 months and after the campaign finish (13 months after the launch) (see figure 4).
