# Supplementary material for: Evaluation and Dissemination of a Checklist to Improve Implementation of Work Environment Initiatives in the Eldercare Sector: Protocol for a Prospective Observational Study
Source: JMIR Res Protoc. 2020 May 13;9(5):e16039. doi: 10.2196/16039 (PMC7254284; doi:10.2196/16039)
Supplement: Multimedia Appendix 2 [file resprot_v9i5e16039_app2.docx]

| **Declaration** |
| --- |

# I hereby certify that the translation of the parts translated in the funding document from the work environment research foundation is true, accurate, and correct "to the best of my knowledge and ability".

Kind regards

Anne Cathrine Tjellesen

Director of Research Coordination
